# In-Vitro Activity of Silybin and Related Flavonolignans against *Leishmania infantum* and *L. donovani*

**DOI:** 10.3390/molecules23071560

**Published:** 2018-06-27

**Authors:** Ana Isabel Olías-Molero, María Dolores Jiménez-Antón, David Biedermann, María J. Corral, José María Alunda

**Affiliations:** 1 Department of Animal Health, Research Group ICPVet, Faculty of Veterinary Medicine, University Complutense, Avda. Puerta de Hierro s/n, 28040 Madrid, Spain; anaolias@ucm.es (A.I.O.-M); mariadolores.jimenez@ucm.es (M.D.J.-A); mariajco@ucm.es (M.J.C.); 2 Institute of Microbiology of the Czech Academy of Sciences, Laboratory of Biotransformation, Vídeňská 1083, 142 20 Prague, Czech Republic; biedermann@biomed.cas.cz

**Keywords:** leishmaniasis, *Leishmania infantum*, *L. donovani*, silybin, dehydrosilybin, dehydroisosilybin, amphotericin, paromomycin, Sb^III^

## Abstract

Flavonolignans from the seeds of the milk thistle (*Silybum marianum*) have been extensively used in folk medicine for centuries. Confirmation of their properties as hepatoprotective, antioxidant and anticancer has been obtained using standardized extracts and purified flavonolignans. Information on their potential effect on *Leishmania* is very scarce. We have investigated the effect of silymarin, silybin and related flavonolignans on the multiplication of promastigotes in vitro and ex vivo on intracellular amastigotes of *L. infantum* (*Li*) and *L. donovani* (*Ld*), causative agents of human and canine visceral leishmaniasis (VL). In addition, the potential synergistic effect of the most active molecule and well-established antileishmanial drugs against promastigotes was explored. Dehydroisosilybin A elicited the highest inhibition against *Ld* and *Li* promastigotes with an approximate IC_50_ of 90.23 µM. This molecule showed a moderate synergism with amphotericin B (AmB) but not with Sb^III^ or paromomycin, although it was ineffective against amastigotes. Antileishmanial activity on intracellular amastigotes of the two diastereoisomers of dehydrosilybin (10 µM) was comparable to that elicited by 0.1 µM AmB. Antiproliferative activity and safety of flavonolignans suggest the interest of exploring their potential value in combination therapy against VL.

## 1. Introduction

Leishmaniasis is a vectorial parasitic disease caused by the infection with *Leishmania* (Protista, Kinetoplastida). Present in all inhabited continents, over one billion people are at risk of leishmaniasis [1] and it is estimated that 14 million people are directly affected [2]. The disease is spectral, from self-healing cutaneous processes, to fatal-unless-treated visceral leishmaniasis (VL), the latter being the second most-lethal human parasitic disease with a 10–20% estimated mortality [3]. Despite national and international efforts, its distribution has actually expanded. Thus, the disease is currently present in previously nonaffected areas due to human migrations, traveling [4], increased distribution of Phlebotominae (Diptera) sandflies vectors as a consequence of climatic change, and the emergence of new target populations in developed countries (e.g., HIV-infected people; recipients of solid organ transplants) [2]. VL is mainly caused by *L. donovani* (*Ld*) and *L. infantum* (*L. chagasi*) (*Li*), the former being anthroponotic (man-to-man transmission) whereas *Li* is zoonotic and infected dogs are the main reservoir. Canine leishmaniasis is very frequent in South America and Europe, particularly the Mediterranean [5], where 2.5 million dogs are infected [6].

Vector control is largely impracticable, human vaccine is not available and immunoprophylaxis of dogs presents several limitations [7]. Chemotherapy, the main tool to limit the extension of the infection, relies on the use of drugs synthesized in some cases over 50 years ago and with important drawbacks such as toxicity, high price of the safer presentations and the appearance of clinical failures/resistances to first-line compounds in endemic areas [8,9]. In this scenario, the exploration of alternative antileishmanial agents is worthy of being pursued. 

Flavonoids are among the most abundant natural compounds of plants acting as growth regulators and providing defense against pathogens. They have been reported as antioxidant, anticancer and neuroprotective agents and have been used as preventives of gastrointestinal and renal disorders, among other indications. Some flavonoids and derivatives have shown activity against Protista such as *Plasmodium* [10] and Trypanosomatids including *Leishmania* and *Trypanosoma* [11,12,13]. 

Flavonolignans are plant secondary metabolites formed by the coupling of a flavonoid moiety with a lignin precursor (phenylpropanoid) [14]. Fruits of *Silybum marianum* (milk thistle) have been used for over 2000 years for hepatic and gall bladder disorders and contain the pharmacologically active extract, silymarin, whose main components are silybin, an equimolar mixture of two diastereoisomers, besides other flavonolignans such as isosilybin, dehydrosilybin, silychristin, silydianin and some flavonoids [15]. Silymarin has been extensively used in human and veterinary medicine [16]. Controversial results obtained have been related to the variable composition of preparations [15] since standardized extracts or individual components have shown anti-inflammatory [17] and hepatoprotective properties (e.g., downregulation of TNF-α) [18]. It has been reported that silymarin has antiproliferative activity against intracellular and extracellular stages of *L. major* (*Lm*) [19] and significantly enhances the in-vitro antiamastigote activity of low doses of meglumine antimoniate against this species [20]. However, information on its potential effect on *Leishmania* causing VL is lacking. 

Our manuscript presents the results obtained in the evaluation of the antileishmanial activity of silymarin and a series of flavonolignans against promastigotes and amastigotes of *Ld* and *Li*, the major causative agents of VL. Moreover, the potential synergy of the most active flavonolignan against the extracellular stage and well-established antileishmanial agents (amphotericin B, paromomycin and Sb^III^) was explored in vitro.

## 2. Results and Discussion

### 2.1. In-Vitro Activity against Li and Ld Promastigotes

**Figure 1:**
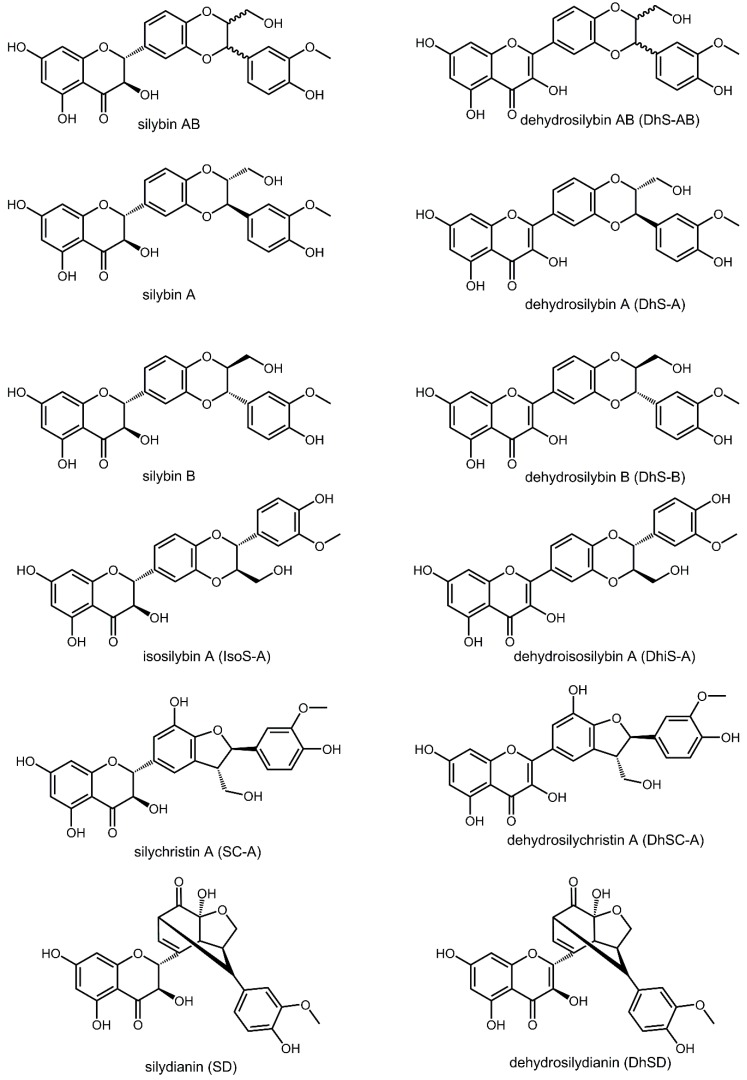
Chemical structures of flavonolignans from *Silybum marianum* (Milk thistle).

An exploratory screening of the antiproliferative activity of a standardized extract, sylimarin, and 12 components (Figure 1), on *Li* promastigotes was carried out. In the range of concentrations employed (from 0.94 µM up to 120 µM), flavonolignans showed notable differences in their antileishmanial activity. Significantly, neither silymarin nor the main component, silybin, elicited any inhibition on *Li* promastigotes. Maximal effect found with dehydrosilydianin, silychristin A, dehydrosilychristin A, dehydrosilybin AB and dehydroisosilybin A was modest and below 50% of the growth of control cultures. The highest inhibitory activity of dehydroisosilybin A against promastigotes was confirmed in *Ld* (Supplementary Material Table S1).

**Figure 2:**
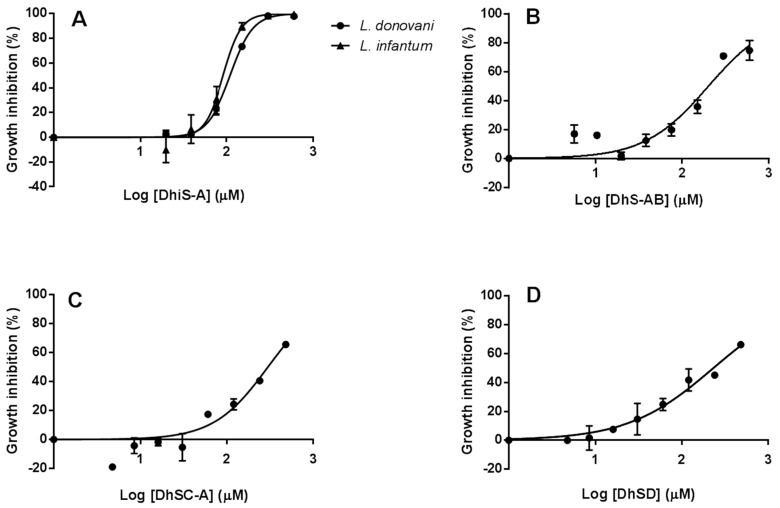
A. Inhibitory effect (%) of different concentrations of dehydroisosilybin A (DhiS-A) on the multiplication of *Li* (UCM9) and *Ld* promastigotes (**A**). Inhibition (%) of the proliferation of *Li* promastigotes by dehyrosilybin AB (DhS-AB) (**B**), dehydrosilychristin A (DhSC-A) (**C**) and dehydrosilydianin (DhSD) (**D**). Concentrations are given as log_x+1_.

Dose–response curves (DRCs) of the molecules displaying the higher activity on *Li* were performed. Cultures were exposed to doubling concentrations from 4.6 µM to 600 µM DhiS-A and DhS-AB; concentration range for DhSC-A and DhSD was from 3.75 µM to 480 µM. For comparative purposes, amphotericin B (AmB), paromomycin (PMM), Sb^III^ and Sb^V^ were included. *Li* and *Ld* strains employed were sensitive to AmB with an approximate IC_50_ of 0.06 µM for both species and they were equally sensitive to PMM. IC_50_ of *Li* for Sb^III^ was ca. 80.6 µM and was insensitive to Sb^V^ (see Supplementary Material Figure S1). There was a clear dose–response relationship although only DhiS-A was able to elicit a complete inhibition of *Li* promastigote multiplication with an IC_50_ of ca. 90.23 µM, and comparable dose–effect response was found for *Ld* (Figure 2A). The other compounds tested were not able to reach inhibition values over 75% with the maximal concentration used (480/600 µM) (Figure 2B–D).

There were species-related variations in the sensitivity to the purified molecules (e.g., silybin AB, silybin B) and as a rule, the *Ld* strain used was more sensitive to the flavonolignans tested than *Li* (see Supplementary Material Table S1). Thus, eight out of the 13 preparations employed inhibited *Ld* promastigote multiplication by ≥30% (120 µM), whereas only three from those elicited a comparable effect on *Li*. These results are in sharp contrast with those previously obtained in *L. major* [19]. Apparently, 25 µM silymarin reduced the multiplication of *L. major* promastigotes by ca. 90% after 72 h, whereas the reduction in Glucantime^®^ (Merial, Lyon, France)-treated cultures (12.5 µM) was significantly lower (ca. 70%). Differences between these results and those obtained by us with *Ld* and *Li* could be related to the differential sensitivity of *Leishmania* species. However, no relationship was found by them between the administered concentrations of the flavonolignans (25 to 100 µM) and the antileishmanial effect observed. Of particular relevance is the actual composition of extracts and silybin since their variable purity is considered the main reason of controversy and uncertainty of results with these compounds [15,21]. Availability of individual diastereoisomers (A, B) of two compounds allowed us to observe that the activity found was not related to the molecular configuration, with similar values of inhibition elicited by isomers and the racemic mixture.

### 2.2. Synergistic Effect of Dehydroisosilybin A and AmB

One of the most favored strategies in the chemotherapy of leishmaniasis is the use of drug combinations with the double purpose of reducing the necessary doses of effective drugs and minimizing the risk of appearance of resistances. Drug–drug interaction was examined with the powerful Chou–Talalay method [22,23]. Table 1 and isobolograms show that DhiS-A and Sb^III^ (1:1), and DhiS-A and PMM (1:5), were antagonists (see Supplementary Materials Figure S2). Mechanistic explanation for the antagonism of the flavonolignan and Sb^III^ is not currently available, although chelation of Fe^II^ and Cu^II^ by dehydrosilybin has been found (unpublished observations). 

**Figure 3:**
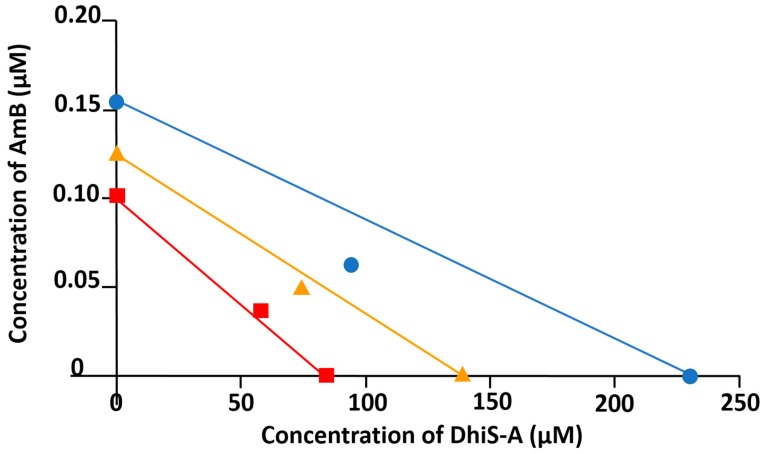
Isobologram of the interaction of AmB with DhiS-A at a fixed concentration ratio (0.01:15). Lines intersect at the x and y axes at concentrations corresponding to EC50 (■), EC75 (▲) and EC90 (●). The same symbols are used for the concentration found for the combination of AmB + DhiS-A to elicit the same effect as the drugs added alone. EC: effective concentration.

The combination of AmB and DhiS-A (0.01:15) showed a more complex interaction. Combination index (CI) values showed antagonism at low concentrations and moderate synergism (CI < 1) at higher concentrations (CI = 0.76 for 95% inhibition) (Table 1, Figure 3). Dose reduction index (DRI) for each molecule in the combination indicated that the amount of DhiS-A could be reduced >4 fold. More importantly, the dose of AmB could be halved to get a 95% inhibition of *Leishmania* multiplication, thus reducing the potential toxicity of the antibiotic (see DRI value and required doses, Table 1).

### 2.3. Efficacy of Flavonolignans against Intracellular Amastigotes

#### 2.3.1. Toxicity of Flavonolignans for Macrophages (Mφ)

Results obtained in the combination studies with promastigotes were encouraging. However, *Leishmania* species have a two-host life cycle, and human and canine infections are caused by the intracellular stage, amastigotes. In the infected hosts, amastigotes multiply within cells from the mononuclear phagocytic system and can show differences with promastigotes in drug sensitivity, or the Mφ can act as barrier or facilitator. Therefore, toxicity of a range of concentrations (from 28.125 to 900 µM) of silybin and related molecules was tested in a surrogate ex-vivo model (mammalian cells: BALB/c mouse peritoneal macrophages, Mφ) (CellTiter-Glo). Dose–response curves showed that molecules displaying the higher toxicity were DhS-AB (IC_50_ ca. 37 µM) and its two isomers A and B (IC_50_ ca. 60 µM and 41.21 µM, respectively), DhiS-A (IC_50_ ca. 30 µM) and DhSC-A (IC_50_ ca. 40 µM). All other molecules had IC_50_ values over 100 µM (see Supplementary Materials Figure S3). These results are in the range of those found for another nondividing mammalian cell, lymphocytes [24].

#### 2.3.2. Efficacy of Flavonolignans against Intracellular Amastigotes

The molecule displaying the highest antiproliferative activity against promastigotes, DhiS-A, was tested at sublethal concentrations (10 µM) against intracellular amastigotes of *Ld* and *Li*. Treatment with the flavonolignan did not affect the Mφ infection (*Li*: nontreated 50.67 ± 4.62% vs. DhiS-A-treated 45.67 ± 7.51; *Ld*-treated: 69.67 ± 8.50% vs. DhiS-A-treated: 67.0 ± 6.08%). Similarly, intracellular burdens (amastigotes/100 Mφ) did not show any reduction after treatment.

**Figure 4:**
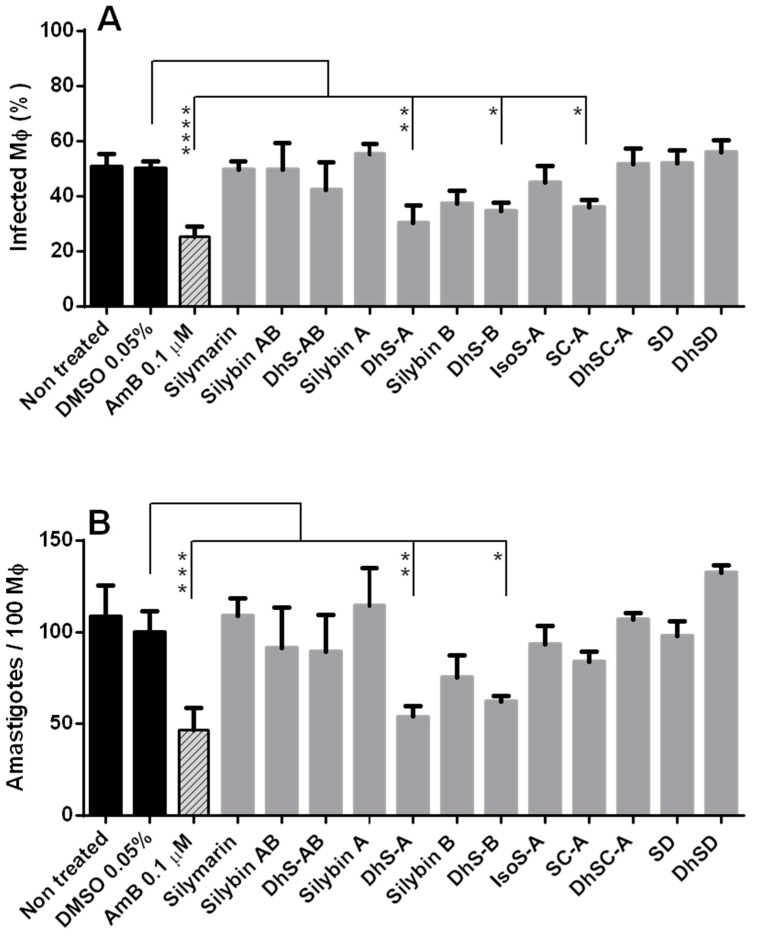
Effect of silymarin and related flavonolignans (10 µM) on the infection (%) of BALB/c mice peritoneal macrophages (Mφ) (**A**) and the number of amastigotes/100 Mφ (**B**) of *Li* BCN150. DhS-AB: dehydrosilybin AB; DhS-A: dehydrosilybin A; DhS-B: dehydrosilybin B; IsoS-A: isosilybin A; SC-A: silychristin A; DhSC-A: dehydrosilychristin A; SD: silydianin; DhSD: dehydrosilydianin. AmB: 0.1 µM amphotericin-treated culture. DMSO 0.05%: control culture. Values given are means ± standard deviation. Significant differences to the DMSO control: * (*p* < 0.05); ** (*p* < 0.01); *** (*p* < 0.001); **** (*p* < 0.0001).

Promastigotes and amastigotes of Leishmania present substantial biochemical and physiological differences. Thus, despite the lack of activity of DhiS-A on the intracellular stage of *Li* and *Ld*, all available flavonolignans were tested against *Li* amastigotes. Treatment with 0.1 µM AmB reduced by 50.0 ± 6.93% the number of Mφ infected and by 57.63 ± 11.47% the number of amastigotes/100 Mφ. The inhibition found with dehydrosilybin A (40.13 ± 12.6% reduction of Mφ infection; over 50% reduction of intracellular amastigotes) for *Li* was not significantly different to the antileishmanial activity of 0.1 µM AmB; this suggests the interest of this molecule for leishmaniasis chemotherapy (Figure 4).

It has been reported that a racemic mixture of silybin inhibited by 53% the multiplication of amastigotes of *L. major* [19], whereas, in our experiment, no antileishmanial activity was found with this compound. Whether or not these differences are factual or related to the methodology used deserves additional research. Interestingly, the molecule eliciting the highest inhibition on promastigotes, DhiS-A, did not show any antiproliferative activity against amastigotes. Several in-vitro and ex-vivo models are used in antileishmanial drug screening including promastigotes, axenic amastigotes and intracellular amastigotes [25], and high-content screening of the intracellular stage has been considered the cellular gold standard for drug discovery [26]. However, with the exception of antimonials, no significant differences of IC_50_ values in promastigotes and amastigotes have been found with the most commonly used antileishmanial drugs (AmB, miltefosine) [27]. Moreover, 26 out of 27 hits found in amastigotes were also identified by promastigotes in a library of bioactive compounds [28]. Tests with promastigotes are less technically demanding and can provide an initial screening, although the stage-related differences found in flavonolignans support the use of intracellular amastigotes. 

Mechanism of action of flavonolignans is not precisely known. Besides their antioxidant role, other potential targets have been indicated including the inhibition and modulation of drug transporters [15,29,30], suppression of cellular inflammation [17], stimulation of protein synthesis [16], and significant inhibitory activity in vitro against *L. major* pteridine reductase I (LmPTR1) [31]. In our case, we found that oxidized stereoisomers displayed a higher toxicity for promastigotes, Mφ and intracellular amastigotes than the parent molecule. These results are in agreement with those obtained in lymphocytes [24] and human hepatocellular carcinoma HepG2 cells exposed to 2,3-dehydroderivatives [32]. Silymarin/silybin is poorly absorbed by the oral route and it is considered very safe [15] since flavonolignans are biotransformed and rapidly excreted. We have found notable differences in toxicity for mammalian cells although, in all cases, toxicity was observed with flavonolignan levels well beyond achievable plasma concentrations after oral administration (ca. 0.8 µM) [33]. Our experiments showed that the required levels of flavonolignans in vitro probably exclude them in monotherapy. However, the activity of some purified components of sylimarin, particularly dehydrosilybin, against *Li* amastigotes ex vivo and the lack of toxicity of flavonolignans suggest that their potential synergy with other antileishmanial drugs for combination therapy should be explored.

## 3. Materials and Methods

### 3.1. *Leishmania* Culture Media, Drugs and Mice Mφ

Two canine isolates of *Leishmania infantum* (*Li*) were used. *Li* UCM9 (M/CAN/ES/2001/UCM9) was originally obtained from a naturally infected dog by the clinical services of the Veterinary Faculty, University Complutense Madrid (UCM). *Li* BCN150 (M/CAN/ES/96/BCN150 zymodeme MON-1) and *L. donovani* (MHOM/SD/43/124) (*Ld*) were obtained from the Instituto de Salud Carlos III (Madrid, Spain). All strains were routinely maintained as promastigotes subpassaged in mid-log phase in medium RPMI 1640 (Lonza, Verviers, Belgium) supplemented with 10% heat-inactivated (56 °C, 40 min) fetal calf serum, 100 U/mL penicillin + 100 µg/mL streptomycin (Lonza), 1% l-glutamine (Lonza) and 1% sterile urine in 5 and 25 cm^2^ culture flasks without filter (TPP, Trasadingen, Switzerland) at 27 °C.

Silymarin was purchased in bulk (from Liaoning Senrong Pharmaceutical, Panjin, People’s Republic of China; batch No. 120501). Flavonolignans isosilybin A, silychristin A (natural silychristin A with about 5% silychristin B) and silydianin were isolated by sephadex LH-20 chromatography as described [34]; silybin AB (natural mixture of silybin A and silybin B ca. 1:1), silybin A and silybin B were prepared as described [35,36]; dehydrosilybin, dehydrosilybin A, and dehydrosilybin B were prepared by oxidation of silybin, silybin A and silybin B with iodine as described [37,38]; dehydrosilychristin A and dehydrosilydianin were prepared by oxidation with molecular oxygen as described [32]. In addition, amphotericin B (AmB) from *Streptomyces* sp., paromomycin sulphate (PMM), potassium tartrate of antimonium III (Sb^III^) (Sigma-Aldrich, Saint Louis, MO, USA) and antimoniate of *N*-methyl glucamine (Glucantime^®^, Merial, Lyon, France) as Sb^V^ were employed.

Mouse Mφ were obtained by RPMI 1640 peritoneal lavage of female BALB/c mice. Mice were purchased from Harlan and housed at the animal facilities of the Instituto de Investigación “Hospital 12 de octubre”, Madrid (Animal facility No. ES280790001164). Experimental design and procedures were approved by the Ethical and Animal Experimentation Committees from the Universidad Complutense and received authorization from the regional government (Ref. PROEX 169/15). Animals were observed daily by qualified animal caretakers. The principles of 3Rs (Replacement, Reduction and Refinement) were applied, and national and international legislation was followed.

### 3.2. In-Vitro Activity against Li and Ld Promastigotes

Mid-log phase promastigotes of *Li* UCM9 (4 × 10^5^ cells/well) were exposed in 96-well microtiter plates (Costar, Corning, NY, USA) to increasing concentrations of the flavonolignans (0.94 µM, 1.87 µM, 3.75 µM, 7.5 µM, 15 µM, 30 µM, 60 µM and 120 µM) in a final volume of 200 µL. Plates were kept under 5% CO_2_/95% air atmosphere at 27 °C for 24 hours. Growth was estimated by the addition of CellTiter-Glo^®^ (Promega, Madison, WI, USA) (1:1) in 96-well microtiter white plates (Costar, Corning). After 10 min incubation (light protected, room temperature), luminescence was determined with FLUOstar Omega (BMG Labtech) reader. Sensitivity of *Ld* was similarly performed with promastigotes exposed to 120 µM. Control cultures (medium, untreated promastigotes, promastigotes treated with 0.2 µM AmB) were included. Experiment was carried out in triplicate and results expressed as % of inhibition of untreated control.

### 3.3. Determination of IC_50_

Approximate IC_50_ values of the flavonolignans showing the highest inhibitory activity against *Li* mid-log-phase promastigotes were determined using dose–response curves. DhSC-A and DhSD were used at 3.75 µM, 7.5 µM, 15 µM, 30 µM, 60 µM, 120 µM, 240 µM and 480 µM; DhiS-A and DhS-AB at 4.68 µM, 9.37 µM, 18.75 µM, 37.5 µM, 75 µM, 150 µM, 300 µM and 600 µM. The compound with the highest activity, DhiS-A, was assayed against *Ld* promastigotes using the latter dilution series and the same culture conditions as above. For comparative purposes, mid-log promastigotes of *Li* and *Ld* were exposed to established anti-*Leishmania* agents: AmB (0.0078 µM, 0.0156 µM, 0.03125 µM, 0.0625 µM, 0.125 µM, 0.25 µM, 0.5 µM and 1.0 µM); Sb^III^ and Sb^V^ (3.75 µM, 7.5 µM, 15 µM, 30 µM, 60 µM, 120 µM, 240 µM and 480 µM) and PMM (4.7 µM, 9.4 µM, 18.7 µM, 37.5 µM, 75 µM, 150 µM, 300 µM and 600 µM). Experiments were carried out in triplicate as above, and approximate IC_50_ values were calculated by nonlinear regression analysis of the dose–response curves with Graphpad Prism 6.

### 3.4. Synergistic Effect between Dehydroisosilybin A, and AmB, Sb^III^ and PMM

Fixed-combination ratios of drugs were based on the IC_50_ values determined for the individual molecules. Mid-log-phase promastigotes of *Li* were exposed for 24 h at constant ratios of 0.01:15 (AmB:DhiS-A), 1:1 (Sb^III^:DhiS-A) and 5:1 (PMM:DhiS-A). Dose–response curves of the combinations were analyzed with the Chou–Talalay method, based on the theorem of combination index (CI)-isobologram and CalcuSyn software [22,23]. This method allows the evaluation of the type of interaction between two molecules according to mass action law. Results can be used to determine quantitatively the synergy (CI < 1), additive effect (CI = 1) or antagonism (CI > 1) of combinations. Moreover, the program allows the calculation of the drug reduction index (DRI): dose reduction in drug combinations to reach a given inhibition compared to the activity of the individual drugs. The software gives other parameters such as the potency (*D*) of the drugs alone or in combination; the shape of the dose–effect curve (*m*) (*m* > 1: sigmoidal curve; *m* = 1: hyperbolic curve; *m* < 1: flat curve) and the linear correlation coefficient representing the conformity of data to the mass action law.

### 3.5. Toxicity of Flavonolignans for Macrophages (Mφ)

Toxicity of flavonolignans for mammalian cells was determined using mouse peritoneal Mφ. Cells obtained were counted and seeded onto 96-well cultures plates (Costar, Corning) at a final concentration of 5 × 10^4^ Mφ/well (100 µL/well). Plates were incubated overnight at 37 °C and humidified 5% CO_2_/95% air to facilitate adherence. Cells were cultured as above for 24 h with different concentrations of the flavonolignans (28.125 µM, 56.25 µM, 112.5 µM, 225 µM, 450 µM and 900 µM). After incubation, cell viability was determined with CellTiter-Glo^®^ as previously described. Untreated and 300 µM AmB-treated control wells were included. Experiments were done in triplicate and approximate IC_50_ determined.

### 3.6. Activity of Flavonolignans against Intracellular Amastigotes

Antiproliferative activity of flavonolignans against intracellular amastigotes was determined ex vivo using mouse peritoneal Mφ. Infections were carried out following the method and modifications described by us [39,40]. Briefly, Mφ were seeded onto 8-well cell culture slides (SLP, Lifesciences Co., Pocheon, Korea) in a final volume of 400 µL (2.5 × 10^4^ Mφ/well). Plates were incubated overnight at 37 °C under humidified 5% CO_2_/95% air atmosphere to facilitate adherence. Stationary-phase promastigotes of *Ld* and *Li* were centrifuged (370× *g*, 10 min) without break in a Ficoll (Sigma, Saint Louis, MO, USA) gradient (0%, 10% in Medium 199 and 30% in sterile phosphate-buffered saline). Metacyclic promastigotes, located in the 10–30% Ficoll interphase, were recovered, washed and opsonized in 15% normal mouse serum in 1:1 solution of RPMI and HBSS medium (Gibco, Life Technologies, Paisley, UK), 0.15 mM CaCl_2_ and 1 mM MgCl_2_ at 37 °C, 5% CO_2_ atmosphere for 30 min. Purified metacyclic promastigotes were added to the Mφ cultures using a ratio 4:1 (promastigotes:Mφ) for *Li* and 10:1 for *Ld*. Plates were incubated at 33 °C overnight as above. Noninternalized promastigotes were eliminated by repeated washing with fresh medium. Treatments (10 µM) were added to the cultures and kept for 24 h. Slides were methanol-fixed and stained with May–Grünwald Giemsa (Merck) to determine the % of infection and the number of amastigotes/100 Mφ under microscope. Cultures untreated, treated with 0.05% DMSO and treated with 0.1 µM AmB were included as controls. Experiments were done in triplicate and the activity was expressed as % of inhibition compared to untreated cultures.

### 3.7. Statistical Analysis

Results given are mean ± standard deviation. Differences between groups were analyzed with 1-way ANOVA (Dunnet’s multiple comparisons test) and the level of significance was set at *p* < 0.05. Calculation of drug–drug interaction and isobolograms were performed with CalcuSyn program. Statistical analysis and figures were done with GraphPad Prism 6 (GraphPad Software, La Jolla, CA, USA).

## 4. Conclusions

Low efficacy and emergence of resistance, and apparent stagnation in the discovery of new chemical entities, support the exploration of natural and naturally derived molecules as potential antileishmanial agents. We have tested the inhibitory activity against *Li* and *Ld*, in vitro and ex vivo, of a standardized mixture of *S. marianum* fruits (silymarin) and 12 components, including the most abundant, silybin. Higher toxicity (mammalian cells, parasites) was observed with oxidized molecules. Dehydroisosilybin A displayed the highest antiproliferative effect on *Leishmania* promastigotes and moderate synergism with AmB. However, this flavonolignan did not show any significant effect on the multiplication of intracellular stages. Maximal activity against amastigotes of *Li* was obtained with 10 µM dehydrosilybin stereoisomers with values comparable to those obtained with AmB (0.1 µM). Since amastigotes are the actual stage causing the disease, our results support the use of amastigotes for further characterization of the potential antileishmanial value of flavonolignans. Safety of flavonolignans and significant inhibition on amastigotes’ multiplication found suggest the interest of exploring their potential value in combination with other currently used antileishmanial drugs on intracellular amastigotes.

## Figures and Tables

**Table 1:** Dose–effect relationship of the combination of antileishmanial drugs (Dn) and dehydroisosilybin A (DhiS-A) on *Li* promastigotes ^1^.

Drug Combination D_n_ + DhiS-A	% Growth Inhibition/ED_n_	CI Values	DRI		Dose Required (µM)	
			*D_n_*	*DhiS-A*	*D_n_*	*DhiS-A*
AmB	10	1.49 (antagonism)	2.85	0.88	0.023	34.79
(0.01:15)	25	1.24 (moderate antagonism)	2.74	1.13	0.030	44.61
	50	1.06 (additive effect)	2.64	1.47	0.038	57.21
	75	0.92 (additive effect)	2.54	1.90	0.048	73.36
	90	0.81 (moderate synergism)	2.39	2.91	0.062	94.08
	95	0.76 (moderate synergism)	2.26	4.28	0.074	111.42
Sb^III^	50	1.89 (antagonism)	0.82	1.50	56	56
(1:1)	75	2.09 (antagonism)	0.92	0.99	141.12	141.12
	90	2.50 (antagonism)	1.04	0.65	355.63	355.63
	95	2.93 (antagonism)	1.13	0.49	666.84	666.84
PMM	50	1.98 (antagonism)	39.54	0.51	822	164
(5:1)	75	1.65 (antagonism)	182.9	0.60	1145	229
	90	1.38 (moderate antagonism)	845.2	0.72	1594	318
	95	1.23 (moderate antagonism)	2393	0.81	1997	399

^1^ Drugs were combined at constant ratios (0.01:15; 1:1 and 5:1) and their dose–effect relationships were assessed by the Chou–Talalay method [22,23] using CalcuSyn software. Combination index (CI) was calculated by the combination index equation. CI of <1, 1 and >1 indicate synergism, additive effect and antagonism, respectively, at different effective doses (ED10, ED25, ED50, ED75, ED90, ED95). Synergistic effect in bold. DRI: fold dose reduction in a drug combination to reach an inhibition level compared to the drug used as a single agent. Computer-simulated dose-required concentrations of each drug to achieve a given effect level are given.

## Supplementary Materials

The following are available online at http://www.mdpi.com/1420-3049/23/7/1560/s1, Figure S1: Dose–effect response of *L. infantum* and *L. donovani* promastigotes to AmB, PMM and Sb^III^; Figure S2A: Isobologram of the interaction of Sb^III^ with DhiS-A at a fixed concentration ratio (1:1); Figure S2B: Isobologram of the interaction of PMM with DhiS-A at a fixed concentration ratio (5:1); Figure S3: Mortality of mouse peritoneal Mφ induced by different concentrations of flavonolignans; Table S1: Effect of flavonolignans (120 µM) on the multiplication of *L. donovani* and *L. infantum* promastigotes.
