# Supplementary material for: In-Vitro Activity of Silybin and Related Flavonolignans against Leishmania infantum and L. donovani
Source: Molecules. 2018 Jun 27;23(7):1560. doi: 10.3390/molecules23071560 (PMC6100512; doi:10.3390/molecules23071560)
Supplement: Supplementary file 1 [file molecules-23-01560-s001.zip › Legend to Figures Supplementary Materials.docx]

**LEGEND TO FIGURES SUPPLEMENTARY MATERIALS:**

**Figure S1**: Dose-effect response of *L. infantum* (A, C, E) and *L. donovani* (B, D, F) promastigotes to AmB, PMM and Sb^III^. Results given as growth inhibition (%) compared to untreated control cultures. Concentrations of antileishmanial drugs given as log_x+1_ transformed µM.

**Figure S2A**: Isobologram of the interaction of Sb^III^ with DhiS-A at a fixed concentration ratio (1:1). Lines intersect at the x and y axes at concentrations corresponding to EC50 (■), EC75 (▲) and EC90 (●). The same symbols are used for the concentration found for the combination of Sb^III^ + DhiS-A to elicit the same effect as the drugs added alone. EC: effective concentration.

**Figure S2B**: Isobologram of the interaction of PMM with DhiS-A at a fixed concentration ratio (5:1). Lines intersect at the x and y axes at concentrations corresponding to EC50 (■), EC75 (▲) and EC90 (●). The same symbols are used for the concentration found for the combination of PMM + DhiS-A to elicit the same effect as the drugs added alone. EC: effective concentration.

**Figure S3**: Mortality (%) of mouse peritoneal Mφ induced by different concentrations (log_x+1_ transformed µM) of flavonolignans. Approximate IC_50_ for each molecule is given. Abbreviations as in Figure 1.
