# Supplementary material for: In-Vitro Activity of Silybin and Related Flavonolignans against Leishmania infantum and L. donovani
Source: Molecules. 2018 Jun 27;23(7):1560. doi: 10.3390/molecules23071560 (PMC6100512; doi:10.3390/molecules23071560)
Supplement: Supplementary file 1 [file molecules-23-01560-s001.zip › Table S1..docx]

**Supplementary Materials**

**Table S1**: Effect of flavonolignans (120 µM) on the multiplication of *L. infantum* and *L. donovani* promastigotes. Values [mean ± standard deviation (SD)] represent the reduction (%) compared to untreated cultures.

|  | **Reduction (%)** | |
| --- | --- | --- |
| **Compound** | ***L infantum*** | ***L. donovani*** |
| Silymarin ^1^ | -0.37±8.79 | 32.19±4.91 |
| Silybin AB | 5.40±2.02 | 36.99±3.91 |
| Dehydrosilybin AB | 17.89±4.33 | 9.01±3.59 |
| Silybin A | -3.18±9.09 | 14.25±7.6 |
| Dehydrosilybin A | 10.19±6.97 | -38.70±5.41 |
| Silybin B | -3.82±9.11 | 30.94±5.02 |
| Dehydrosilybin B | 20.94±11.63 | 38.06±2.90 |
| Isosilybin A | 6.94±6.43 | 30.65±5.85 |
| Dehydroisosilybin A | 38.30±4.96 | 87.62±2.82 |
| Silychristin A | 27.11±8.64 | 19.17±5.75 |
| Dehydrosilychristin A | 30.61±3.33 | 46.74±1.24 |
| Silydianin | 16.43±8.63 | 20.47±3.08 |
| Dehydrosilydianin | 42.19±5.42 | 29.83±2.17 |

^1^ Silymarin concentration tested is 57.89 µg/mL
