# Supplementary figures and images for: In-Vitro Activity of Silybin and Related Flavonolignans against Leishmania infantum and L. donovani
Source: Molecules. 2018 Jun 27;23(7):1560. doi: 10.3390/molecules23071560 (PMC6100512; doi:10.3390/molecules23071560)

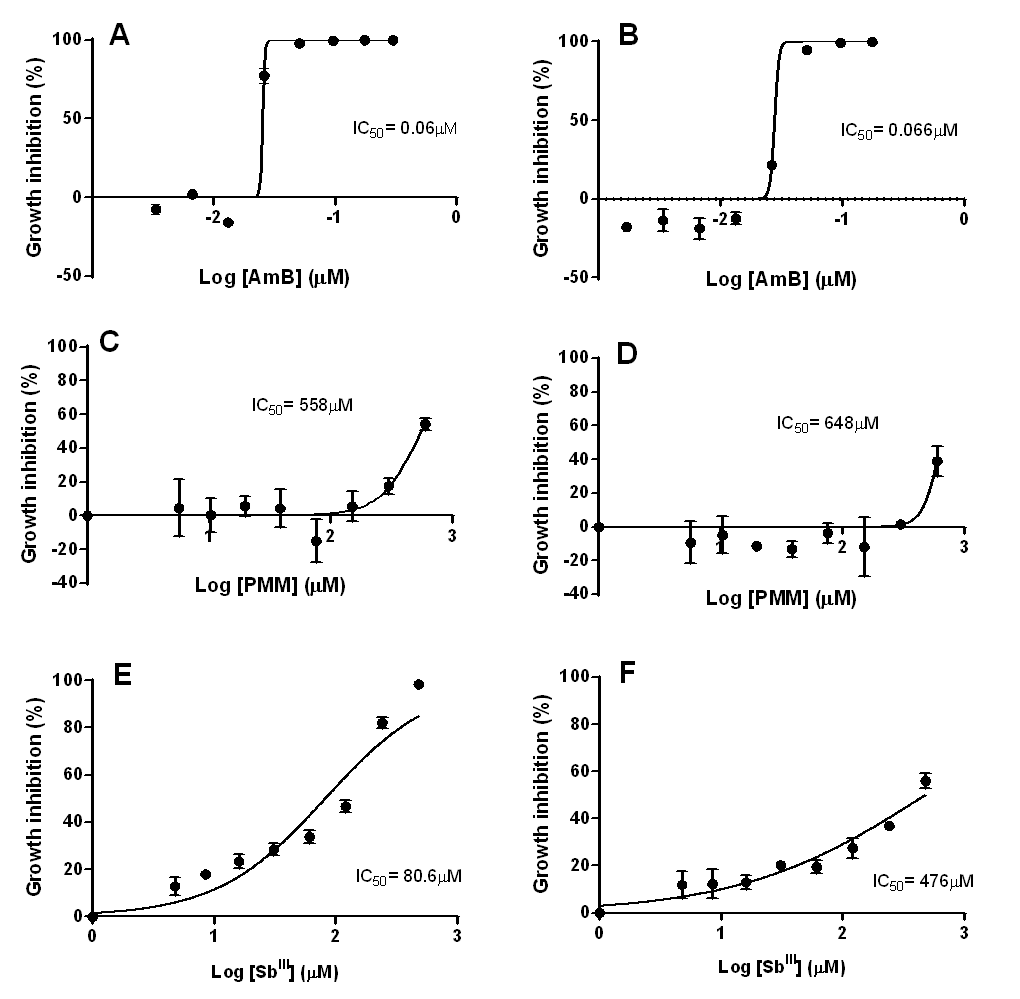

Supplement: Supplementary file 1 [file molecules-23-01560-s001.zip › Figure S1.tif]

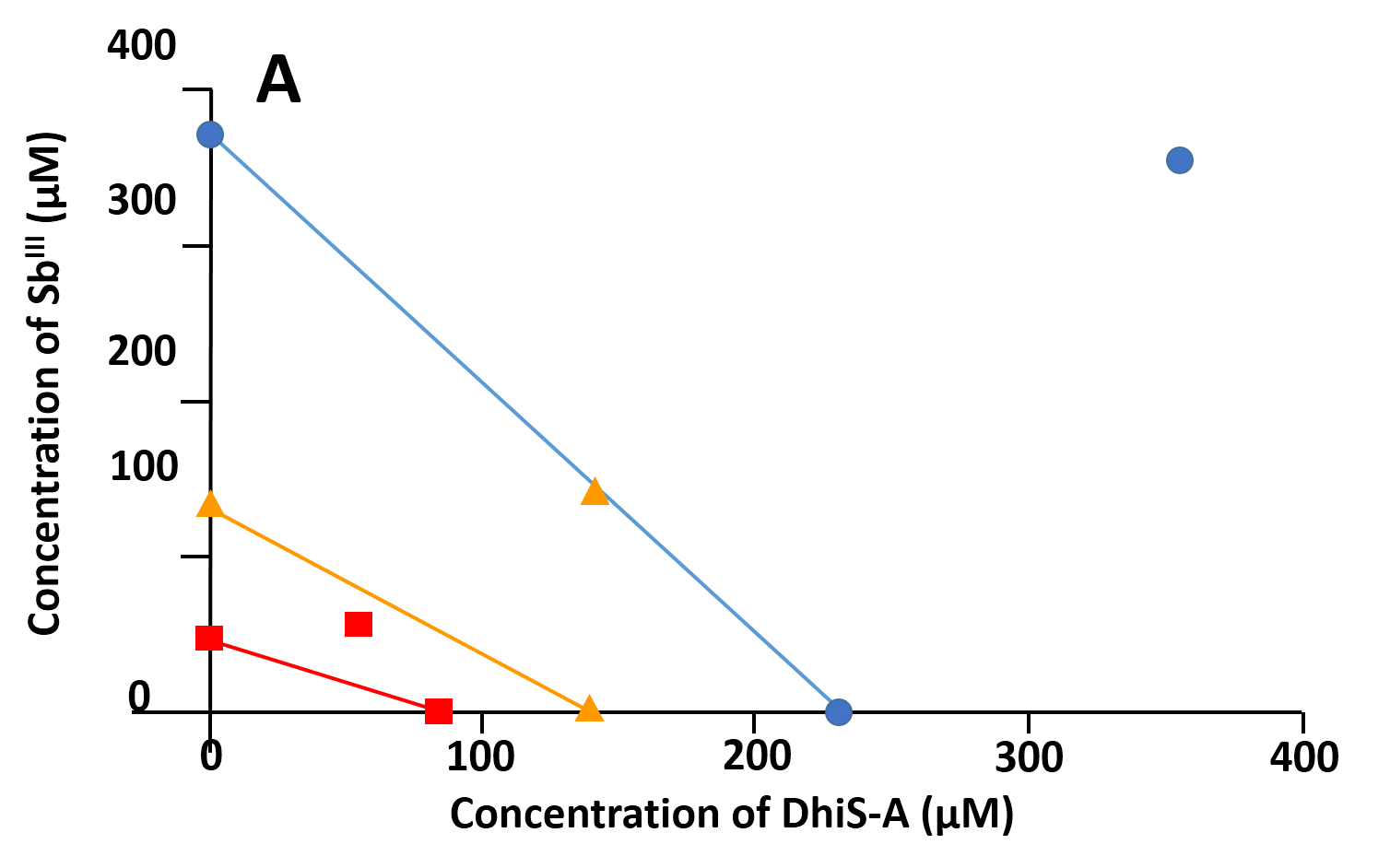

Supplement: Supplementary file 1 [file molecules-23-01560-s001.zip › Figure S2A.tif]

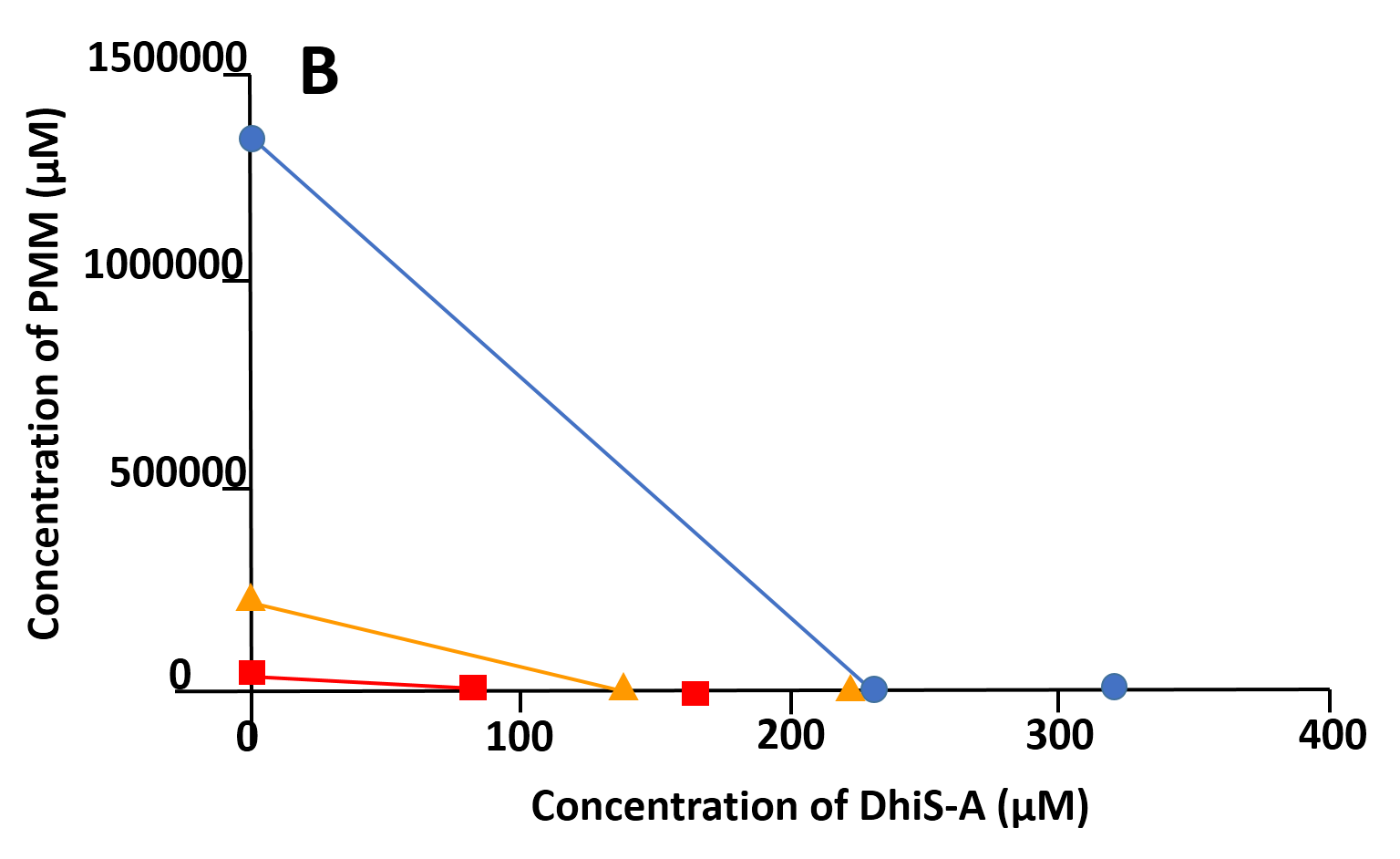

Supplement: Supplementary file 1 [file molecules-23-01560-s001.zip › Figure S2B.tif]

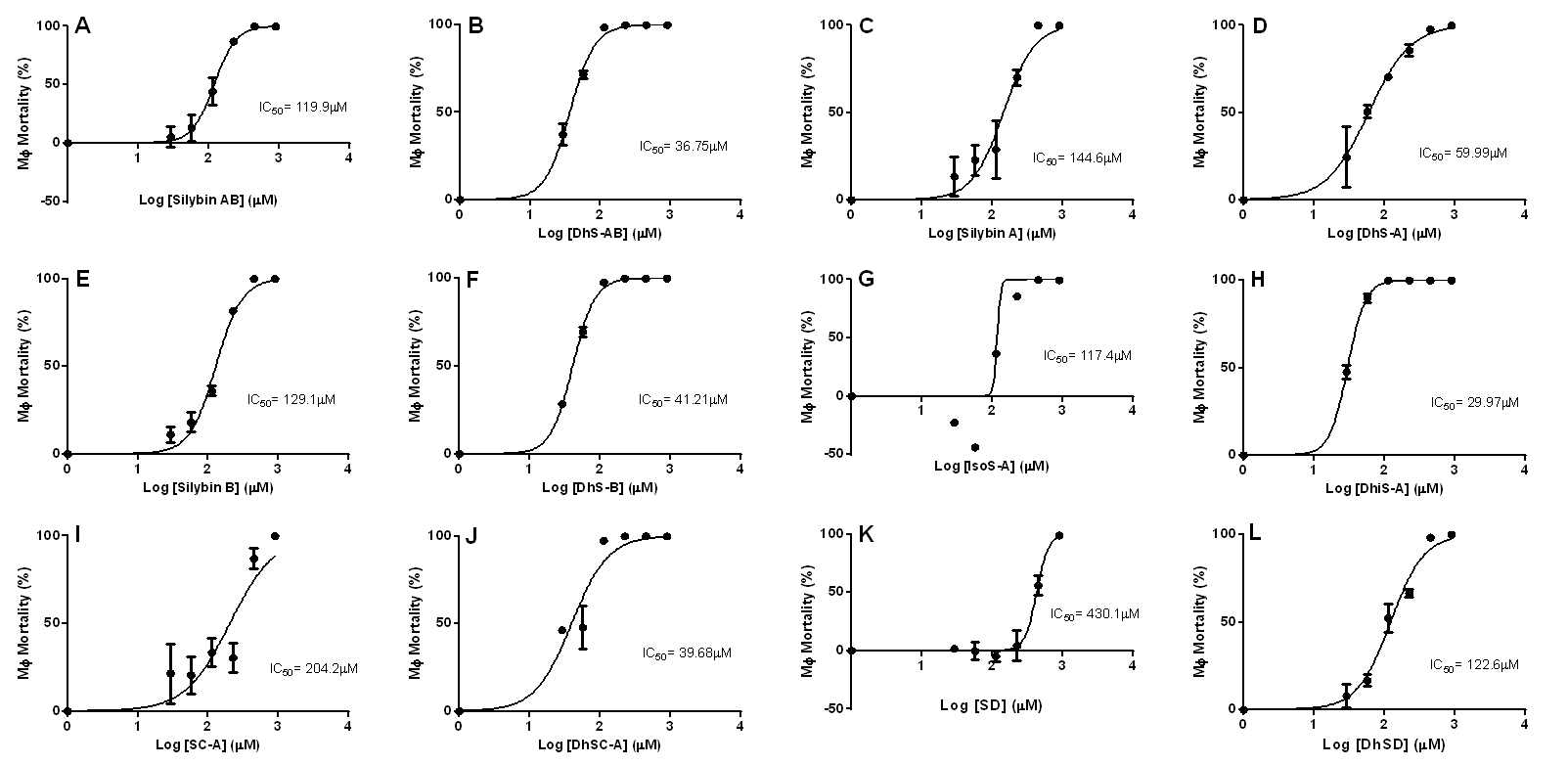

Supplement: Supplementary file 1 [file molecules-23-01560-s001.zip › Figure S3.tif]
